# The Accuracy of Prehospital Fluid Resuscitation of Burn Patients: A Systematic Review

**DOI:** 10.3390/ebj3040044

**Published:** 2022-11-10

**Authors:** Fahad Alsaqabi, Zubair Ahmed

**Affiliations:** 1 Emergency Medical Care Department, College of Applied Medical Sciences, Imam Abdulrahman Bin Faisal University, Dammam 34212, Saudi Arabia; 2 Institute of Inflammation and Ageing, University of Birmingham, Edgbaston, Birmingham B15 2TT, UK; 3 Centre for Trauma Sciences Research, University of Birmingham, Edgbaston, Birmingham B15 2TT, UK

**Keywords:** prehospital, burn, fluid, resuscitation, emergency medical services

## Abstract

Early management of burns is an essential component of achieving desirable patient outcomes. One of the earliest points of patient management in the case of burn injuries is in the prehospital setting. Unlike first aid, which can be provided by a non-healthcare worker, fluid resuscitation can be provided in the prehospital setting by emergency medical services personnel. This systematic review aims to investigate whether burn patients are receiving accurate fluid resuscitation in the prehospital setting. In addition, it will investigate if existing inaccuracies could impact patient outcomes negatively. This systematic review was completed in accordance with the guidelines from the Preferred Reporting Items for Systematic Reviews and Meta-Analyses (PRISMA). The search for eligible studies started by searching relevant databases (PubMed, Embase, Medline, and Google Scholar). The selected studies were screened, and data were extracted and analyzed using a narrative synthesis approach. Seven studies met the inclusion criteria of this review, with a total of 961 patients. All seven studies included in this review reported that the volume of fluids for resuscitation purposes received by burn patients in the prehospital setting was inaccurate. However, most reported that the patient outcomes were not affected. Most of the studies were rated as “good,” however, and further high-quality randomized control studies are required before strong recommendations can be made.

## 1. Introduction

Early management of burns is an essential component of achieving desirable patient outcomes, with fluid resuscitation being one of the most important components of burn management [1]. One of the earliest points of patient management in the case of burn injuries is in the prehospital setting. In fact, a large number of patients that arrive in hospitals suffering from burn injuries have been transported by emergency medical services (EMS) [2]. Unlike first aid, which can be provided by non-healthcare workers limited to basics like irrigation and bandaging, fluid resuscitation can be provided in the prehospital setting by EMS personnel [2]. However, there has been evidence to suggest that prehospital management of burn patients suffers from inaccurate total body surface area (TBSA) estimates, where prehospital EMS personnel are not as accurate with their TBSA estimates as burn surgeons or emergency department physicians who are considered to have the most accurate TBSA estimates [3,4]. This has the potential to impact the treatment of burn patients when it comes to intravenous fluid resuscitation, which is one of the most important treatments to be established as early as possible to increase the chances of patients’ survival [1].

One of the most commonly used formulae for resuscitating burn patients is the Parkland formula, which is dependent on the TBSA to deliver accurate volumes of fluids over a specific period of time [5]. This raises the question of whether prehospital EMS personnel are delivering accurate volumes of fluids to burn victims under their care during transport to the hospital as part of their prehospital care protocol [3,6,7,8,9]. This systematic review aims to investigate whether burn patients are receiving accurate fluid resuscitation in the prehospital setting. In addition, it will investigate if inaccuracies existed and whether they negatively impacted patient outcomes. The population of interest in this review is burn patients who received fluids resuscitation in the prehospital setting before being admitted to a burns unit or a burns center for specialized care, regardless of the type of prehospital setting, i.e., military versus civilian and mode of transportation, i.e., via ground ambulance versus air transport. However, for the purposes of this review, the focus will be on fluids being administered to patients before reaching the aforementioned target destinations. Cooling, irrigation, and other burn treatments were not considered for the purposes of this review. Fluid volumes administered to patients were compared to local protocols in the studies selected for this review and were compared to reach a conclusion. The outcome of comparing all selected studies will be to answer the main question posed in this systematic review.

## 2. Materials and Methods

### 2.1. Data Sources and Searches

This systematic review was completed in accordance with the guidelines from the Preferred Reporting Items for Systematic Reviews and Meta-Analyses (PRISMA) [10]. The search for eligible studies was conducted on 1 July 2022 by searching relevant databases (PubMed and Embase were searched through the Ovid interface, and Medline was also searched using the Ovid interface and Google Scholar). After a few test runs to narrow down some effective search term combinations, the same Boolean search terms of choice were used to search these databases (prehospital OR pre-hospital OR out of hospital AND EMS OR emergency medical services OR ambulance) AND (Fluid resuscitation OR fluid AND care AND management) AND (burn OR burns OR burn injury AND thermal injury). Although, it should be mentioned that in the case of searching through Google Scholar, a few of the aforementioned terms were combined to narrow down the search results.

### 2.2. Study Selection

For this systematic review, a set of inclusion criteria were established to identify eligible studies to include for reviewing. These criteria are as follows: studies must be relevant to the Patient/population, intervention, comparison and outcomes (PICO) framework [11] of this review; studies must state the results of prehospital fluid resuscitation protocols; studies were not age-restricted to extreme age groups, i.e., pediatric (<18 years) and geriatric patients (>65 years). Conference abstracts that met the inclusion criteria were included. Studies were excluded if they were not relevant to the PICO framework of this study, had unavailable full text, were age restricted to extreme age groups, or were not available in English.

All studies were imported to Endnote and then exported to Rayyan.ai for duplicate removal and to use the blind feature so both researchers could independently make a decision before the data extraction phase. The justification for not including extreme age groups is that fluid resuscitation in pediatric patients usually involves a specifically different or modified formula for that population and might not reflect the overall accuracy of prehospital fluid resuscitation. However, the case with geriatric patients is different, as they tend not to do as well as other patient groups [4] and hence may disproportionally sway the overall results in favor of poor patient outcomes.

### 2.3. Data Extraction

Studies retrieved from the literature search were initially screened using their title and abstract by 2 independent reviewers (F.A. and Z.A.). Subsequently, relevant studies underwent full-text analysis to determine eligibility, and any disagreements were resolved through discussion. Data extraction was then performed, where the following information was extracted onto a spreadsheet: study characteristics (first author, year, title, study design, country, inclusion and exclusion criteria, intervention and control protocols, outcome measures, and results), population characteristics (sample size, age, gender, wound location [upper or lower limb]), and outcome measures. Where information was unavailable, the tables were left blank, as no assumptions were made. The primary outcome extracted for this systematic review was prehospital fluid resuscitation accuracy. Secondary outcomes included the effect of inaccuracies on patient outcomes.

### 2.4. Quality Assessment

The NIH Quality Assessment Tool for Observational Cohort and Cross-Sectional Studies (https://www.nhlbi.nih.gov/health-topics/study-quality-assessment-tools, last accessed on 22 July 2022) was selected to assess the risk of bias of each study. The tool consisted of 14 questions to be answered with yes, no, or other (CD, cannot determine; NA, not applicable; NR, not reported) according to what was reported in each study. “Yes” was given a value of 1, while “no” and “other” were given a value of 0. Studies that scored a total of 0–5 were given a “poor” rating, 6–9 were considered “fair,” and 10–14 were rated as “good.” Both researchers involved in this review used the tool independently of each other. The results were then compared, and if there were any disagreements on the scoring, the matter was resolved by discussion.

### 2.5. Data Synthesis

Due to the heterogeneity in the included studies, a meta-analysis was not possible. Instead, it was decided that a narrative synthesis was the right way to synthesize and summarize the data gathered from these studies. This began by searching for common themes between the included studies. Then, the themes were reviewed and approved by both authors (F.A. and Z.A.). This created a basis on which results from all studies can be compared, and a comprehensive conclusion can be drawn from them. The themes were as follows: all burn patients were looked at in terms of their need for fluid resuscitation, regardless of the type or site of the burn, as long as the fluid resuscitation was performed in the prehospital setting (military and civilian) and before transport to the hospital.

The accuracy of fluid resuscitation was calculated in each study by the amount of fluid received prior to arrival at a burn’s unit as a percentage of that required according to the burn’s unit’s own assessment of the percent TBSA burn. Since each study used different resuscitation protocols, the accuracy of fluid resuscitation was judged by the same protocol by the local burn unit. “Inaccurate resuscitation” was given as a result if patients were over- or under-resuscitated, i.e., if they received less or more fluid volumes than the needed amounts. Patients that did not receive any resuscitation at all, when they should have according to the local protocol as reported in the study, were deemed as under-resuscitation and therefore labeled as “inaccurate resuscitation.”

While this was performed to reach a conclusion for resuscitation accuracy, the same steps were not followed to reach a conclusion about the reported patient outcomes in the included studies. This is mainly because all 5 studies that reported patient outcomes reported their results in a similar manner. Two of the studies, however, did not report any patient outcomes. The synthesis summary was performed by one researcher and then reviewed and approved independently by the second reviewer to minimize reporting bias.

### 2.6. Certainty Assessment

The grading of recommendations, assessment, development and evaluation (GRADE) tool was adapted to assess the certainty of evidence in this review. However, it should be noted that the tool was originally developed to work with a narrative summary of the results, as it is usually used in conjunction with meta-analysis results. The domains assessed were risk of bias, which was assessed by using the NIH Quality Assessment Tool for Observational Cohort and Cross-Sectional Studies (https://www.nhlbi.nih.gov/health-topics/study-quality-assessment-tools, last accessed 22 July 2022), Imprecision was assessed by the overall information size of all included studies and the reported results, inconsistency was assessed by evaluating consistencies and similarities between magnitudes of effects of all included studies, indirectness, which was assessed by evaluating whether populations, interventions and comparators gave related evidence to the question raised by the review, and publication bias, which was assessed by how comprehensive the studies selection process was and if only studies with positive results were published.

## 3. Results

**Figure 1:**
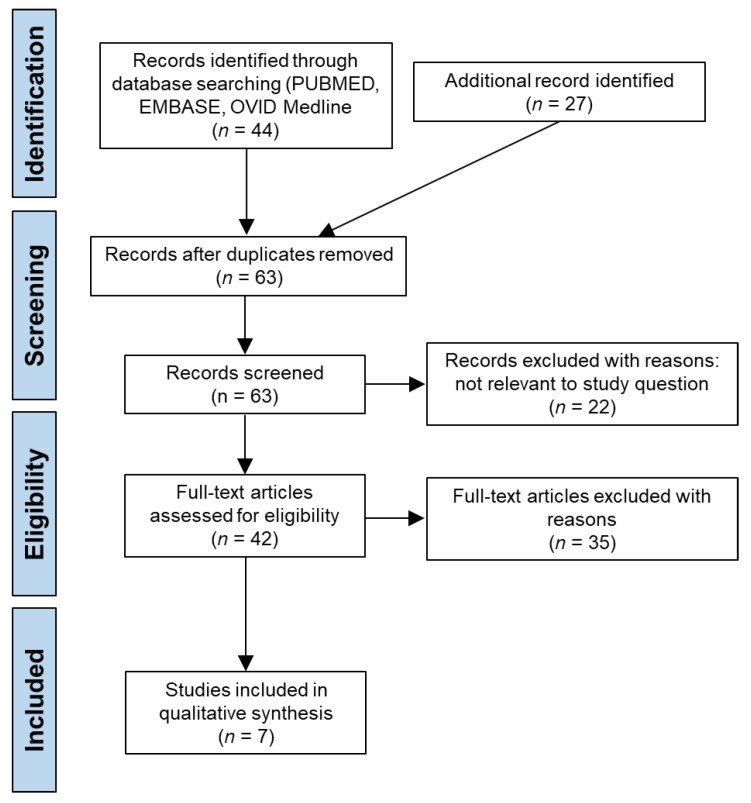
PRISMA flowchart of the search strategy.

The search for eligible studies was conducted between 1 July 2022 and concluded on 22 July 2022. Figure 1 shows the PRISMA flowchart of the study selection process.

PubMed yielded 130 articles, of which 19 were selected for screening. Embase was searched through the Ovid interface and yielded 503 articles, of which 13 were selected for screening, and Medline yielded 399 articles, of which 20 were selected for screening. Google Scholar yielded 17,900 articles, a number of articles that was not practical to explore. However, the first 20 pages were investigated and resulted in the inclusion of 19 articles. The reference sections of the included articles were checked for any possible inclusions but resulted in no further articles. The full-text versions of all selected articles were screened, which resulted in the exclusion of 64 articles (eight were duplicates, 22 were not relevant to the research question, 33 did not include fluid resuscitation data, and one was not in English). Finally, only seven studies were selected as meeting all requirements and were included for further analysis in this systematic review [12,13,14,15,16,17,18].

### 3.1. Characteristics of the Studies

The selected studies spanned a publication period from 1999 to 2020. Two studies were conducted in the United Kingdom [12,17], two in Europe (Finland and Switzerland) [16,18], one in the United States of America [13], one in Australia [14], and one in Iraq [15]. Three studies did not have age restrictions in the included sample [12,17,18], while three involved adults only [14,15,16]. All seven studies were cohort studies (five retrospective [12,14,15,17,18] and two prospective [13,16]). Only one of the studies was in a military setting [15], while the remaining six were in a civilian setting [12,13,14,16,17,18]. The total sample size of all studies was 961 patients. Table 1 summarizes the characteristics of the included studies.

### 3.2. Risk of Bias in Studies

**Figure 2:**
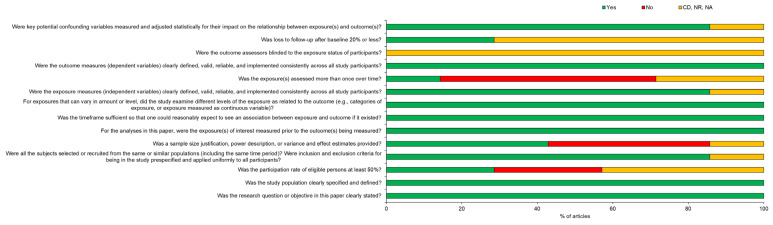
Risk of bias assessment summary. CD = cannot determine, NR = not reported, NA = not applicable.

Two out of the seven studies were graded as fair [13,17], while the other five were graded as good [12,14,15,16,18]. One out of 14 questions was answered with “Not applicable” across all seven studies (Figure 2). This question relates to the blinding aspect of the study. However, due to the nature of the included studies, we believe that this would not affect the quality of the studies. Only four questions were answered with “Yes” across three studies or less. At the same time, nine questions were answered with “Yes” across at least six categories (Figure 2).

### 3.3. Accuracy of the Volume of Fluids Given

All seven studies included in this review reported that the volume of fluids received by burn patients in the prehospital setting prior to in-hospital treatment was inaccurate [12,13,14,15,16,17,18]. Most studies reported that the average inaccuracies were due to over-resuscitation, while two studies reported that inaccuracies occurred due to under-resuscitation. Table 2 summarizes the results of the accuracy of fluid resuscitation in the selected studies.

One study compared paramedics and prehospital physicians and found that inaccuracies persisted, with prehospital physicians tending to deliver excess fluids compared to paramedics (185% compared to 169%, respectively) [16]. Another compared the accuracy of fluid resuscitation using two different protocols, one following the Parkland formula and one following the new Alfred formula. The study found that inaccuracies persisted during the use of both protocols [14]. Similarly, another study also explored the topic by comparing two different protocols. However, in this study, both protocols were used at the same time, one to calculate the volumes needed to resuscitate with human albumin solution (HAS) and the other with crystalloids (the modified Muir and Barclay formula and the Parkland formula, respectively). The study found inaccurate resuscitation on average using both protocols, with over-resuscitation being the most common inaccuracy [12].

In a different study, where patients were being transported by air, 52% of these patients received inaccurate volumes of fluids, with more than 90% being over-resuscitated [13]. The only study that explored military burn casualties noted that the majority of patients did not receive any fluid resuscitation (58.3%), but those who received intravascular fluids were over-resuscitated according to the Committee for Tactical Combat Casualty Care (CoTCCC) protocols [15]. In another study investigating whether prehospital burn management standards were met or not, only 33% of patients received accurate fluid resuscitation [17]. The last study looked at both small and large burns and found that, on average, there were inaccuracies in prehospital fluid resuscitation. Furthermore, it found that small burns tended to be over-resuscitated and large burns tended to be under-resuscitated [18].

### 3.4. Patient Outcomes after Receiving Inaccurate Resuscitation

Although none of the studies presented outcome data, five [12,14,16,17,18] out of the seven studies stated that significant changes in patient outcomes after incorrect fluid resuscitation, such as 30-day mortality rate, incidence of escharotomy, laparotomy and intubation status, were not observed (Table 3). This suggests that the overall risks of incorrect resuscitation to patient outcomes are negligible, but problems associated with fluid overload, for example, may be unlikely, except perhaps in elderly patients. However, this is an area that needs more work to reach a definitive conclusion. Nevertheless, all of the studies reported that incorrect fluid resuscitation occurred as a result of inaccurate burn size estimation by prehospital staff as compared to estimation by the burns unit. This, on occasion, led to some patients receiving up to 20 times the calculated amount [12].

### 3.5. Certainty Assessment

Due to the fact that all included studies were cohort studies in design, it was acknowledged that these types of studies are not high on the reliability of evidence hierarchy. Nonetheless, the evidence provided was subjected to a certainty of evidence assessment. The GRADE tool was used to assess the certainty of the evidence, and the results are as follows:

#### 3.5.1. Risk of Bias

After using the NIH Quality Assessment Tool for Observational Cohort and Cross-Sectional Studies, it was possible to rate the overall risk of bias for all studies. Since only two out of seven studies received a rating of fair while the rest received a rating of good, it is believed by the authors (F.A. and Z.A.) that the overall risk of bias is not affected to a degree that negatively impacts the studies. However, due to the fact that three items in the risk of bias tool used had negative results across most of the studies, this lowered the risk of bias slightly. Nonetheless, the overall bias rating is still believed to be in the positive range to borderline moderate.

#### 3.5.2. Imprecision

Even though the overall population was above 900 patients, most studies did not report confidence intervals (CIs) for the results of fluid resuscitation. Therefore, precision cannot be determined for the overall results. Thus, the decision was made to consider the precision of the evidence as imprecise.

#### 3.5.3. Inconsistency

The results were consistent in reporting inaccuracies in the average amounts of prehospital fluid resuscitation across all the included studies, and most studies reported no change in patient outcomes due to this. Due to the effect being constant across most studies, the evidence was deemed consistent.

#### 3.5.4. Indirectness

The target population, intervention, and comparators all align with the question raised by this review. All interventions explored were about prehospital fluid resuscitation, administered to burn patients, and were compared to local protocols where the studies took place. Therefore, the evidence was considered direct and in line with the direction taken by this review.

#### 3.5.5. Publication Bias

Publication bias is not believed to be a factor since studies with negative results are published, including all the selected studies. Furthermore, the study selection process followed by this review was thorough.

#### 3.5.6. Large Effect

Since all studies have reported inaccurate resuscitation and most reported no change to patient outcomes, this led to the belief that the effect is overwhelming any possible bias that is common to cohort studies. As alluded to earlier, observational studies are considered low-level evidence. Therefore, they start with a low rating on the certainty of evidence assessment. In our case, the overall certainty was brought down to “very low” due to the fact that imprecision cannot be determined. However, a large effect was observed, and therefore, the evidence was upgraded back to low.

## 4. Discussion

This systematic review sought to investigate the accuracy of IV fluid resuscitation delivered by prehospital EMS personnel to burn patients in the prehospital setting and prior to transfer to ED or burn centers. The results from systematically reviewing seven studies revealed that, on average, prehospital resuscitation of burn patients was not aligned with protocols. The studies included in this review shed light on the different situations where these accuracies persisted. The different protocols used for fluid resuscitation made no difference to potential errors in fluid resuscitation, in which patients were most often given fluids beyond the recommended guidelines and hence were over-resuscitated. Surprisingly, however, all of the studies were in agreement that these errors did not impact patient outcomes such as 30-day mortality or other parameters. Under four different guidelines (Parkland, the modified Muir and Barclay, and the Alfred formulae and the (CoTCCC) protocols), prehospital resuscitation, albeit in varying degrees, was inaccurate [12,14,15]. This raises the question of what issue is causing these inaccuracies to occur.

The level of education did not seem to have an effect in one study, as it showed that both prehospital physicians and paramedics are over-resuscitating patients. In fact, physicians, who have higher levels of education, were more likely to inaccurately administer more fluids than paramedics [16]. With evidence suggesting that the level of education is not the culprit, others have explored the possibility of the TBSA estimates being the issue. Being TBSA-dependent, fluid resuscitation was shown to be impacted by inaccuracies in estimating TBSA in both adults [19] and children [3,20]. In the studies included in our systematic review, TBSA estimation by prehospital or A&E staff appeared to be significantly different from that of burn center specialists and hence was the single biggest reason for errors in estimating fluid resuscitation volumes. According to burn center specialists, over-resuscitation averaged from 50% to over 150% of the calculated percentage of fluid used to resuscitate patients [12,13,14,15,16,17,18]. In general, overestimation of TBSA by prehospital or A&E staff was the main reason for errors in fluid resuscitation and was independent of the guidelines used to generate resuscitation volumes. This suggests that there is a need for an easier or faster way to estimate the TBSA in the prehospital setting to help EMS personnel manage their patients without sacrificing fluid resuscitation accuracy.

Despite these inaccuracies in fluid resuscitation volumes, it did not seem to affect patient outcomes in the long run or show any side effects. Given the fact that over-resuscitation was the most commonly seen inaccuracy among the included studies, fluid creep was not reported to be a side effect in any of the reviewed studies, despite the fact that in some studies, patients received seven to 20 times the calculated volume [12,13,14,15,16,17,18]. This side effect, however, has been shown to appear with overloading patients with IV fluids within the first 24 h [21]. This is probably due to the fact that once patients have arrived at a specialist burn center, correct TBSA estimation would have taken place, and any remedial treatments offered to patients and hence burn centers are potentially compensating for the inaccurate fluid resuscitation by prehospital and A&E staff. None of the studies also reported a side effect specific to under-resuscitation, even though hypovolemia is one of the most commonly seen medical conditions in burn patients [22]. An interesting perspective to follow in future study designs might be to include patients that were transferred directly to burn centers, where one would assume that correct volumes of fluid resuscitation would be used. Following the outcomes of these patients might be an interesting perspective in terms of overall recovery from burns.

### 4.1. Limitations

This review identified key issues with the prehospital resuscitation of burn patients. However, there are certain limitations to this review. The study design of the included studies is one of the main limitations, and all the included studies are longitudinal cohort studies by design that depended on collecting data from databases or one regional healthcare facility. Although no major bias was detected in these studies, they remain low-level evidence, and this review would have provided a higher level of evidence if randomized controlled trials were available in the literature. Another limitation is the heterogeneity of how the results are reported in the studies included in this review, which made it difficult to attempt a meta-analysis with the data provided. Along with randomized controlled trials, a meta-analysis would greatly improve the level of evidence to come out of this review. The fact that only two reviewers were involved in the selection of studies to include in this review, and the fact that only English articles were included, made a limitation to this review, as there might have been studies that were missed during the selection process. Furthermore, the in-hospital intervention being a continuation of the prehospital management might have corrected any side effects that might have been associated with the inaccuracies that occurred in the prehospital setting.

### 4.2. Recommendations

The findings of this systematic review demonstrate that errors in calculating the percent TBSA were the single biggest reason for inaccurate burn resuscitation volumes given to patients. Continuous training and education in TBSA calculations are required to minimize their errors. Specific courses such as those of the British Burn Association Emergency Management of Severe Burns (https://emsb.org.uk/, last accessed 30 October 2022) should assist in better management of burns patients. In addition, further studies exploring the same topic are needed, as most of the available publications suffer from having small sample sizes or from being low-evidence cohort studies. Future studies should focus on randomization and sample size to be able to precisely determine a relationship between prehospital resuscitation and the overall patient condition before arriving at the target facility and receiving specialized care; and ways to improve the accuracy of burn resuscitation in the prehospital setting.

## 5. Conclusions

Overall, this systematic review identified key points to answer the question at hand. Firstly, prehospital fluid resuscitation of burn patients is inaccurate on average. These inaccuracies persisted regardless of the different protocols explored (Parkland, the modified Muir and Barclay, and Alfred formulae and the (CoTCCC) protocols), setting type (civilian versus military), education level (physician versus paramedic), and mode of transportation to the receiving facility. Secondly, over-resuscitation is found to be the most common inaccuracy. Lastly, there no evidence was found that prehospital fluid resuscitation inaccuracies had any direct effects on the overall outcomes of patients suffering from burn injuries. Correcting these inaccuracies, however, and conducting more RCTs should be the focus of future studies.

## Figures and Tables

**Table 1:** Characteristics of the included studies.

Study	Study Location	Setting Types	Study Population	No. of Patients	Study Design	Patient Description
Collis et al. [12]	UK	Civilian	Adults and children	256	Retrospective	Patients transferred to the Regional Burns Unit from 1994 to 1996
Shewakramani et al. [13]	USA	Civilian	NS	100	Prospective	NS
Mitra et al. [14]	Australia	Civilian	Adult	126	Retrospective	Patients with a TBSA burnt of >/= 20% and admitted between 1 July 1999 and 30 June 2009
Lairet et al. [15]	Iraq	Military	Adult	48	Retrospective	U.S. casualties >20% total body-surface-area (TBSA) thermal burn
Kallinen et al. [16]	Finland	Civilian	Adult	67	Observational	Adult (≥18 years) burn patients transported directly to Hospital with TBSA ≥ 20%, needing mechanical or assisted ventilation
Ashman et al. [17]	UK	Civilian	Adults and children	278	Retrospective	Oral scalding-burns-major burns-electrocution
Maudet et al. [18]	Switzerland	Civilian	Adults and children	86	Retrospective	All patients suffering from thermic burn and treated at the University Hospital Burn Centre, regardless of age

Notes: NS = not specified; TBSA = total body surface area.

**Table 2:** Accuracy of the volume of fluids given.

Study	Accuracy of Resuscitation Method Compared to Standard Protocol
Collis et al. [12]	Inaccurate (55% over-resuscitation, 21% under-resuscitation, 24% accurate)
Shewakramani et al. [13]	Inaccurate (52% received inaccurate fluid resuscitation)
Mitra et al. [14]	Inaccurate (only 39.86% received accurate resuscitation)
Lairet et al. [15]	Inaccurate (58.3% did not receive resuscitation, 41.7% over-resuscitated)
Kallinen et al. [16]	Inaccurate (volumes of 185% administered in the prehospital physician-treated groups, 169% in the paramedic-treated group)
Ashman et al. [17]	Inaccurate (of this cohort, only 33% received the necessary IV fluids)
Maudet et al. [18]	Inaccurate (seven-fold over recommended fluid levels in the small burns subgroup, infused volume nearly corresponded with the recommendations in the large burns subgroup)

**Table 3:** Patient outcomes after receiving inaccurate resuscitation.

Study	Effect of Inaccuracies on Patient Outcome
Collis et al. [12]	Did not affect patient outcome
Shewakramani et al. [13]	NS
Mitra et al. [14]	Did not affect patient outcome
Lairet et al. [15]	Did not affect patient outcome
Kallinen et al. [16]	Did not affect patient outcome
Ashman et al. [17]	NS
Maudet et al. [18]	Did not affect patient outcome

Notes: NS = not specified.

## Data Availability

The data presented in this study is found within the article.
